# Orthodontic biomechanics with intermaxillary elastics

**DOI:** 10.1590/2177-6709.28.3.e23spe3

**Published:** 2023-07-24

**Authors:** Marcel Marchiori FARRET

**Affiliations:** 1 Centro de Estudos Odontológicos Meridional (CEOM), Especialização em Ortodontia (Passo Fundo/RS, Brazil).; 2 Private practice (Santa Maria/RS, Brazil).

**Keywords:** Orthodontics, Malocclusion, Angle Class II, Orthodontics, corrective

## Abstract

**Introduction:**

Intermaxillary elastics are orthodontic resources widely used in various malocclusions. Their main advantages are low cost, easy insertion and removal by patients, and application versatility. As main disadvantages, we can highlight the need for cooperation from patients and the side effects normally present in treatments with this resource. Knowledge of the biomechanics involved in the use of intermaxillary elastics is essential to take full advantage of the desired effects and avoid unwanted effects in their use.

**Objective:**

Therefore, the objective of this article is to describe the anchorage preparation, connection methods, time and force of use, and side effects involved in the use of intermaxillary elastics for the treatment of anteroposterior, vertical and transverse problems. For that, clinical cases and biomechanics schemes will be presented, in which all these details will be described.

## RESUMO

**Introdução:**

Os elásticos intermaxilares são recursos ortodônticos amplamente utilizados nas diversas más oclusões. Possuem como principais vantagens o baixo custo, fácil inserção e remoção pelos pacientes, e versatilidade de aplicação. Como desvantagens podemos destacar a necessidade de cooperação dos pacientes e os efeitos colaterais normalmente presentes nos tratamentos com esse recurso. O conhecimento da biomecânica envolvida no uso dos elásticos intermaxilares é fundamental para se obter o máximo de efeitos desejados e evitar os efeitos indesejados na sua utilização.

**Objetivo:**

O objetivo desse artigo é descrever o preparo da ancoragem, as formas de conexão, o tempo e a força de uso, além dos efeitos colaterais envolvidos na utilização dos elásticos intermaxilares para tratamento de problemas anteroposteriores, verticais e transversais. Para isso, serão apresentados casos clínicos e esquemas de biomecânica em que todos esses detalhes serão descritos.

## INTRODUCTION

Intermaxillary elastics are removable materials used in most orthodontic treatments, such as aids in the correction of anteroposterior, vertical, or transverse problems.^1-5^ They have been used since the end of the 19^th^ century, and the materials and application techniques have evolved considerably over time.^5^ ^-^ ^9^ The elastics were originally made only with latex, which are still the most used elastics today. However, synthetic orthodontic elastics are currently being produced, being an option in situations in which patients have allergic reactions to latex.^3^ ^,^ ^8^ ^,^ ^10^ ^-^ ^13^

Elastics have several advantages over other orthodontic resources, such as: low cost; are removable, facilitating feeding and hygiene; easy insertion and removal by patients; esthetically acceptable in most applications; and great versatility in orthodontic biomechanics.^4^ ^,^ ^9^ ^,^ ^14^ The main disadvantages of elastics include: need for the patient’s cooperation, degradation in the oral environment, side effects such as root resorption, occlusal plane asymmetries, allergic reactions and dental rotations, among others.^2^ ^,^ ^4^ ^,^ ^6^ ^,^ ^7^ ^,^ ^9^ ^,^ ^15^ ^,^ ^16^ The need for significant cooperation on the part of patients, as well as the usually present side effects, are the main reasons for the replacement of elastics with skeletal anchorage in some cases. However, they are still widely used in orthodontic treatments for being a more conservative choice, for their versatility in biomechanics, and due to their low cost.^9^ ^,^ ^16^ ^,^ ^17^

The use of elastics may seem simple from the viewpoint of installation and removal, and when the orthodontist only considers the desired effects of the mechanics. However, knowledge of the side effects involved and how to control them is essential for the success of treatments that use these materials.^8^ ^,^ ^14^ ^,^ ^18^ ^-^ ^20^ Therefore, based on the frequency of intermaxillary elastics use in orthodontic treatments and the need for knowledge about the correct indication, biomechanical application, and knowledge of the desired and undesirable effects, the purpose of this article is to conduct a review on the main types of intermaxillary elastics used in orthodontics associated with case reports and explanations using biomechanical schemes.

## TYPES OF INTERMAXILLARY ELASTICS

### ANTEROPOSTERIOR (AP)

» Class II or Class III (bilateral).
» Class II or Class III (unilateral).
» With sliding iig (Class II or Class III).

### VERTICAL (INTERCUSPATION)

» Class I, Class II or Class III (bilateral posterior).
» Class I, Class II or Class III (unilateral posterior).
» Anterior.

### TRANSVERSAL

» Crossbite.
» Inverse crossbite.
» Midline.

## CLASS II ANTEROPOSTERIOR ELASTIC

Class II elastic is possibly the most used intermaxillary elastic in the orthodontic routine. It has an essentially horizontal direction, and its objective is to move the upper teeth posteriorly and the lower teeth anteriorly, correcting an existing Class II relationship. In addition to the traditional indication for Class II sagittal relationship correction, it can also be used to assist loss of lower posterior anchorage or retraction of maxillary anterior teeth in the presence of diastemas or spaces created by extractions. It can also be used in situations of decompensation for orthognathic surgery in Class III cases.^2^ ^,^ ^4^ ^,^ ^7^ ^,^ ^9^ ^,^ ^16^ ^,^ ^18^ ^,^ ^21^ ^,^ ^22^

## ANCHORAGE PREPARATION

**Figure 1:**
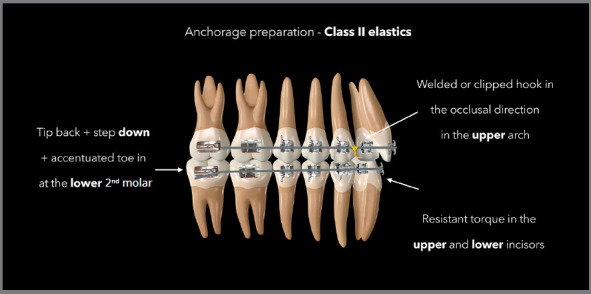
Illustration describing the anchorage preparation required in rectangular stainless steel archwires for the use of Class II elastics.

When using Class II elastics with the aim of moving a group of teeth, it is recommended to prepare both arches with alignment and leveling up to the rectangular stainless steel wires, preferably 0.019 × 0.025-in, in order to reduce unwanted effects.^8^ ^,^ ^9^ It is possible to reduce the thickness of some arches when greater effects are desired on that arch; however, it should be done knowing that the side effects also tend to be accentuated. In addition, it is recommended to perform tip-back bends with step-down and accentuated toe-in on the lower second molar to avoid mesial inclination, extrusion, and rotation of that tooth, which will serve as support for the elastic. In lower incisors, when it is desired to minimize the projection, a buccal root torque should be included, which is a torque that is resistant to unwanted movement. In the maxillary arch, the torque of the incisors must be increased to avoid uprighting of these teeth.^4^ ^,^ ^9^ ^,^ ^15^ Figure 1 demonstrates how to prepare the anchorage for the use of Class II elastics.

## CONNECTION

The elastics must then be connected to the lower second molars, in order to make the resultant forces more horizontal compared to situations in which the elastic is connected to the first molars or more anterior teeth of the lower arch.^8^

In the upper arch, the ideal is to use welded or clipped hooks in the region between the lateral incisor and canine, preferably facing downwards (occlusal), which also favors the formation of a more horizontal resultant. Another alternative is to make bends in the wire, such as loops or deltas, to connect the elastics to the upper arch. Direct connection to canine brackets should be avoided because the resultant will be more vertical and can promote greater effects on this tooth and not on the entire arch, consequently promoting rotation and distal tipping of the canines crowns and opening of spaces between the canines and lateral incisors.

## SIZE, FORCE, AND WEAR TIME

The most suitable for this application are the 1/4-in, 5/16-in, or 3/8-in medium strength elastics, distributing an average of 200gf on each side.^4^ ^,^ ^6^ ^,^ ^8^ ^,^ ^19^ Initially, during the period we call the active phase, they should be ideally used for 24 h, being removed only for food and hygiene. When the desired results are obtained, we move on to the retention phase that should last two months on average, using the same size of elastic, but with light force and still for 24 hours a day. Finally, it is also possible to carry out the post-retention phase, as a guarantee of maintaining the results obtained. At this stage, the light elastic should be used for 12 hours a day for one month.^15^ ^,^ ^23^

## SIDE EFFECTS

**Figure 2:**
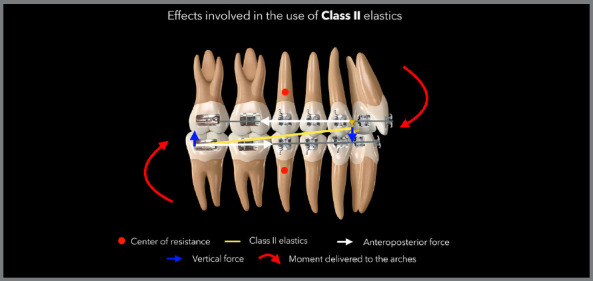
Illustration demonstrating the side effects commonly observed with the use of Class II elastics.

**Figure 3:**
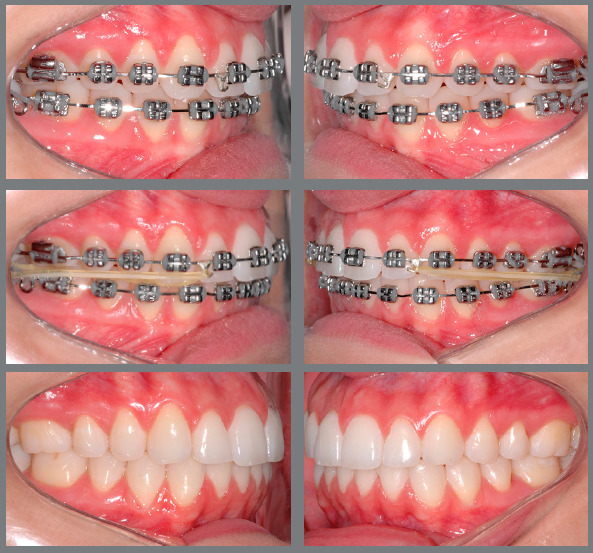
Patient treated with Class II 5/16-in medium force elastics. The elastics were used bilaterally, in the final phase of the treatment, to correct a Class II relationship of 2 mm.

The use of Class II intermaxillary elastics always leads to side effects, which are effects that occur alongside the main effects and may or may not be referred to as undesirable effects, depending on each case. These side effects include clockwise rotation of the occlusal planes (pitch axis), an increase in the vertical dimension due to extrusion of the lower molars, uprighting of the maxillary incisors, proclination of the mandibular incisors, distal tipping of the maxillary posterior teeth, mesial tipping of the mandibular posterior teeth, and root resorption (Fig 2).^2^ ^,^ ^7^ ^,^ ^14^ ^-^ ^16^ ^,^ ^19^ ^,^ ^20^ ^,^ ^24^ Biomechanical side effects can be eliminated or minimized by using rectangular arches, preferably stainless steel 0.019 × 0.025-in or thicker, when even more control is desired in one of the arches.^4^ ^,^ ^8^ ^,^ ^9^ In addition, specific adjustments in certain regions of the arch are also fundamental in relation to this objective of controlling side effects, as shown in the diagram of the Figure 1. Figure 3 shows a case in which Class II elastics were used at the end of the treatment to correct small AP discrepancy. In situations where side effects have to be totally eliminated, the use of skeletal anchorage is suggested as a substitute for the use of intermaxillary elastics, with intra-arch mechanics. Another alternative is to use the skeletal anchorage device as a lower posterior connection for the Class II elastic. In this case, the side effects in the lower arch will be eliminated, however, in the upper arch they will be similar to those found in conventional biomechanics with Class II elastics.

## CLASS III ANTEROPOSTERIOR ELASTIC

Class III elastic has as main aim to move the upper teeth mesially and the lower teeth distally, and it is very similar to the Class II elastic regarding the anchorage preparation for use, wear time, force, and control of side effects - however, with inversion of the arches. It can be indicated in cases where it is desired to increase the loss of anchorage in the upper arch or retraction in the lower arch when there are diastemas or spaces created by extractions. It is also used in cases of surgical decompensation in a Class II patient, when it is desired to increase the overjet by proclination of the maxillary anterior teeth or uprighting of the mandibular anterior teeth. It can also be used as an alternative to obtain space in the lower arch, avoiding or reducing the projection of the lower incisors during alignment and leveling.^4^ ^,^ ^6^ ^,^ ^7^ ^,^ ^17^

## ANCHORAGE PREPARATION

**Figure 4:**
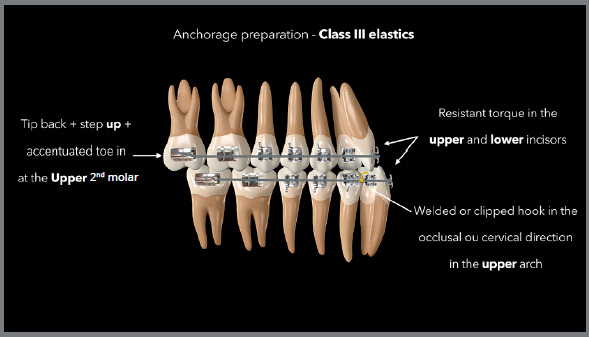
Illustration describing the anchorage preparation required in rectangular stainless steel archwires for the use of Class III elastics.

The diagram in Figure 4 demonstrates how to prepare the anchorage required to use Class III elastics. To distalize the entire lower arch associated with mesialization of the entire upper arch, it is also necessary to prepare both arches with alignment and leveling up to rectangular steel wires, preferably 0.019 × 0.025-in.^23^ ^,^ ^24^ To control side effects, tip-back with step-up and accentuated toe-in bending can be performed on the upper second molar to avoid mesial inclination, extrusion, and rotation of this tooth that will serve as support for the elastic. To avoid excessive uprighting in the lower incisors, a lingual torque must be included, whereas the torque must be reduced in the upper incisors, working as a buccal torque, both being considered resistant to side effects.^4^

## CONNECTION

Class III elastics should be attached to the maxillary second molars, when present and included in the archwire, and attached to hooks welded to the lower arch in the region between the lateral incisor and canine.^4^ ^,^ ^6^ ^,^ ^8^ In the case of Class III elastic, when the overbite allows, the hook in th lower arch can be positioned occlusally, forming a more horizontal resultant. If the overbite does not permit, the hook can be positioned cervically. As with the Class II elastic, connection directly to the canines should be avoided.

## SIZE, FORCE, AND WEAR TIME

Elastic sizes commonly used in the Class III direction are 1/4-in, 5/16-in, or 3/8-in medium force, applying an average of 200gf on each side.^6^ ^,^ ^19^ ^,^ ^24^ The same protocol of active, retention, and post-retention phases must be followed, as described in Class II elastics.

## SIDE EFFECTS

**Figure 5:**
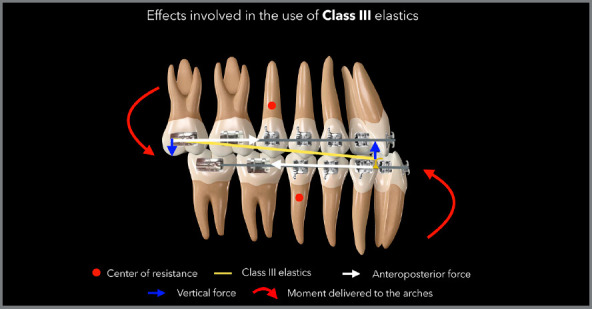
Illustration demonstrating the side effects commonly observed with the use of Class III elastics.

The use of elastics with a Class III correction direction will also promote important side effects. These side effects include counterclockwise rotation of the occlusal planes (pitch axis), increased vertical dimension due to extrusion of the maxillary molars, uprighting of the mandibular incisors, projection of the maxillary incisors, distal tipping of the mandibular posterior teeth, and mesial tipping of the maxillary posterior teeth (Fig 5).^7^ ^,^ ^17^ ^,^ ^20^ ^,^ ^23^ ^-^ ^27^ Patients with Class III malocclusion usually have reduced and increased display of the maxillary anterior and mandibular anterior teeth when smiling, respectively. The tendency with the use of Class III elastics is to rotate the planes counterclockwise, which will further reduce exposure of the maxillary anterior teeth. Therefore, in these cases, prolonged use of Class III elastic should be avoided, and maximum control of side effects should be ensured. Alternatives such as skeletal anchorage, orthognathic surgery, or the use of auxiliary appliances, such as the headgear associated with a Class III elastic, should be considered in these cases, when it is observed that the esthetic results may be unsatisfying at the end of the treatment with the use of Class III elastics and rotation of the planes.

Side effects or unwanted effects can be eliminated or minimized with rectangular arches, 0.019 × 0.025-in steel, or thicker when even greater control is desired in one of the arches. In addition, specific adjustments in certain regions of the arch are also essential in relation to this objective of controlling side effects, as shown in the diagram in Figure 4. When side effects need to be avoided, skeletal anchorage should be used as a substitute for the use of intermaxillary elastics, with intra-arch mechanics. Another possibility is the use of the skeletal anchorage device as an upper posterior connection for the Class III elastic. This situation is indicated when the skeletal anchorage device was installed to perform another movement, for example, upper posterior intrusion. The same device can then be used with superior support for the Class III elastic, eliminating unwanted side effects in the upper arch, leaving only the side effects in the lower arch.

**Figure 6:**
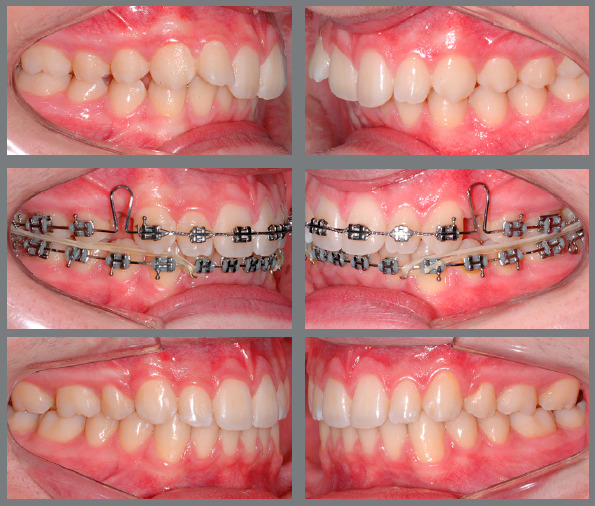
Patient treated with Class III 5/16-in elastics of medium force. In this case, Class III elastics were used as a resource to increase the loss of anchorage in the upper arch after extraction of the first premolars, and as a resource to improve the inclination of the lower incisors that were proclined before treatment, facilitating the finishing procedures with correct overjet and overbite.

Figure 6 reports a case with upper first premolar extractions and the use of Class III elastics to increase anchorage loss in the posterior region of the upper arch and improve the position of proclined lower incisors.

## UNILATERAL CLASS II OR CLASS III ANTEROPOSTERIOR ELASTICS

Anteroposterior (AP) elastics can be used unilaterally when we want to correct an asymmetry in one or both arches. However, this type of elastic configuration has other side effects when used bilaterally, in addition to those already mentioned here.^23^ ^,^ ^28^ It is therefore recommended to use reduced time in these cases, a maximum of three months, and the association of elastics on the opposite side, as described below.^28^

**Figure 7:**
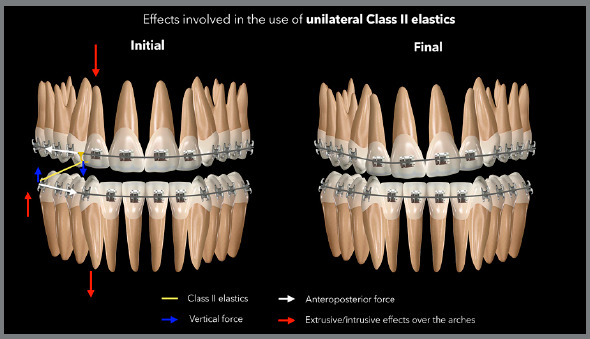
Side effects normally observed with the unilateral use of Class II elastics.

**Figure 8:**
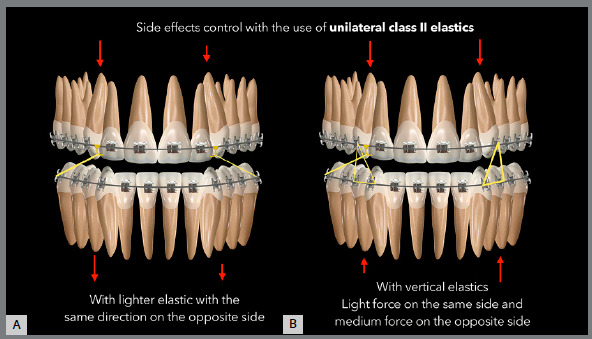
Illustration demonstrating the two possibilities of reducing side effects in situations in which a greater unilateral gain is needed in the Class II correction: Elastic association with the same direction but reduced force on the opposite side (A), and the association of vertical elastics in the canine region, with greater force on the left side, where there is no need for AP correction (B). In the first situation, there is still a greater tendency to extrusion on the right side of the upper arch, as shown by the arrows in red. In the second situation, the extrusive force is similar on both sides of the upper arch; however, there is a greater extrusive force on the left side of the lower arch. To minimize the effects on the lower arch, thicker rectangular archwires can be used.

**Figure 9:**
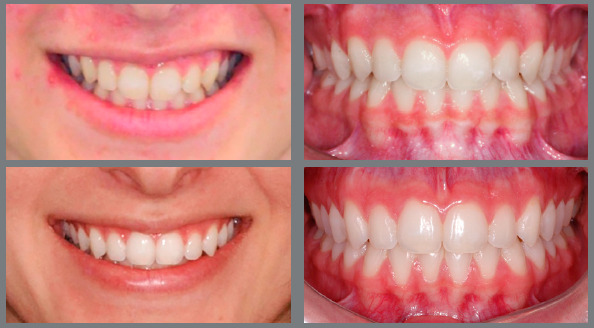
Patient treated with unilateral Class II elastic on the left side, without control of side effects, promoting cant of the upper occlusal plane ( roll axis ) and need for retreatment to level the plane by means of intrusion with a miniscrew, followed by extrusion of the lower arch with vertical elastics.

**Figure 10:**
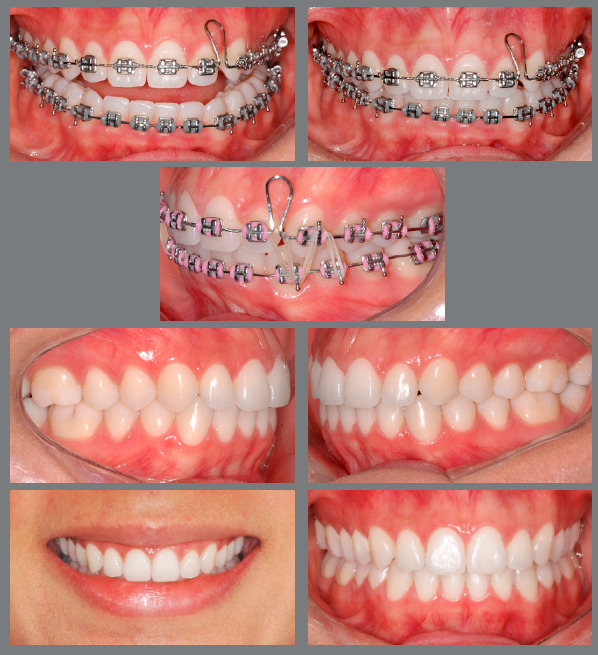
Patient treated with unilateral Class II elastic in the final phase of treatment, promoting cant of the lower occlusal plane ( roll axis ) by extrusion of the posterior region and intrusion of the anterior region on the left side. Vertical elastic was then used on the left side, associated to an asymmetric curve in the archwire to level the lower plane. The upper arch required slight extrusion on the left side, so the extrusive effect was not considered adverse, as it promoted the leveling of the upper arch.

The most common side effect in these situations is the inclination of the occlusal plane in frontal view (roll axis) due to the extrusion force occurring unilaterally, especially in the anterior region of the upper arch when a Class II elastic is used, and in the anterior region of the lower arch when a Class III elastic is used. To avoid this type of side effect, an elastic can be incorporated in the same direction, but with reduced force on the opposite side when a small correction in the AP direction is desired, or else a vertical elastic can be added to the opposite side when the sagittal relationship is already in Class I.^7^ In situations of prolonged use of unilateral elastics (more than three months), it is also possible to observe unilateral extrusion in the posterior region where the elastic is connected. The diagram in Figure 7 presents the side effects usually associated with the use of a unilateral Class II elastic. Figure 8 shows alternatives for the use of unilateral elastics, with reduced side effects. Figures 9 and 10 show cases in which the Class II elastic was used unilaterally for a long period, without controlling for side effects.

## SLIDING JIG

**Figure 11:**
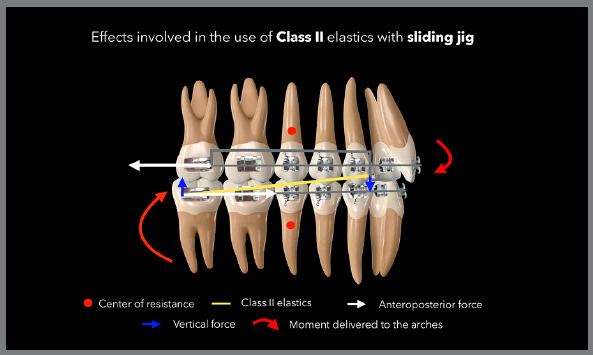
Illustration showing the effects involved in biomechanics with unilateral Class II elastic associated with sliding jig. The extrusive effect in the anterior region is minimized and, mainly, the effect of asymmetric anterior contraction of the arch is eliminated, as the distalization force is moved from the anterior region to the posterior region of the arch.

**Figure 12:**
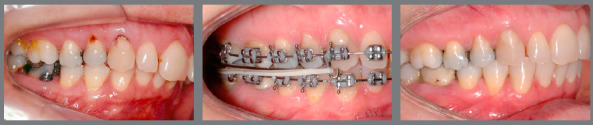
Patient treated with a unilateral Class II elastic associated with a sliding jig. In this case, the patient chose not to use skeletal anchorage or to extract a premolar; so, to minimize side effects, mechanics with a sliding jig was used for tooth-by-tooth distalization and correction of the Class II, subdivision right. To minimize the effects on the lower arch, a 0.021 x 0.025-in rectangular arch was initially used and later a 0.022 x 0.028-in arch was used. The space between teeth #12 and #13 would be closed by means of a composite restoration.

One way to minimize the side effects of using unilateral or bilateral AP intermaxillary elastics is incorporating cursors, as a sliding jig.^4^ ^,^ ^8^ The sliding jig minimizes the extrusive force in the anterior region by having a more horizontal connection, compared to conventional elastics, and by distributing the distalization force of the intermaxillary elastic to the posterior region, thus minimizing the tendency toward asymmetric contraction and cant of the occlusal plane of the arch to be distalized, both in Class II and Class III.^4^ ^,^ ^8^ Figure 11 presents the scheme for using the Class II unilateral intermaxillary elastic associated with the sliding jig, demonstrating how there is a reduction in side effects in the anterior region of the upper arch. Figure 12 shows a case treated with Class II elastic only on the right side, which was associated with a sliding jig, distalizing tooth by tooth.

## VERTICAL ELASTICS (INTERCUSPATION)

Vertical elastics are used to improve the vertical intercuspation relationship between the upper and lower teeth. Like AP elastics, they lead to both desired effects and side effects that may or may not be desired.

## ANCHORAGE PREPARATION

As with the use of AP elastics, the preparation of one or both arches is necessary for the correct use of vertical elastics, for the control of side effects. It is recommended to use a rectangular steel archwire, preferably 0.019 × 0.025-in, to control the torque of the posterior and anterior teeth.^4^

## CONNECTION

When vertical elastics are being used, the connection can normally be made in two ways: directly on the brackets, when they already have hooks; or connection on hooks clipped to the arches, positioned between two brackets. Normally, the connection to clipped hooks present a lower risk of side effects; however, these effects can also be controlled in the connection to the brackets using metallic ties or elastic bands in X shape on these brackets.^4^ It is possible to make Kobayashi-type hooks in brackets that do not have hooks.

## SIZE, FORCE, AND WEAR TIME

The definition of force for vertical elastics is limited because of the difficulty in measuring this force in the mouth. However, there are formats and thicknesses predefined in articles and books for using these elastics in different formats, respecting the rule that an intermaxillary elastic must be stretched to approximately three times its original size to perform its function optimally.^3^ ^,^ ^4^ Examples are the use of 1/8-in medium force elastics for triangles in the canine region, 3/8-in light or medium force elastics for the M configuration, and 3/16 or 1/4-in medium force elastics for the configuration of triangles with Class II or Class III direction, as shown in the diagrams below.

## SIDE EFFECTS

**Figure 13:**
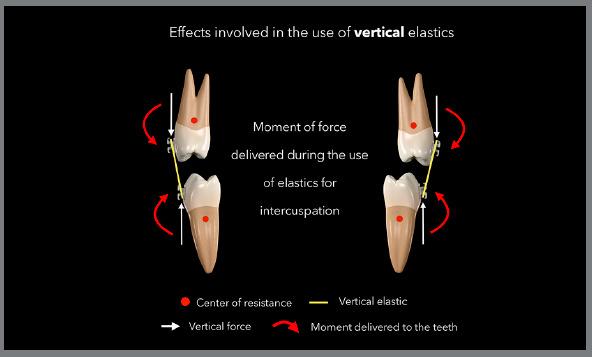
Illustration demonstrating the side effects normally observed with the use of vertical elastics for intercuspation in the posterior region.

**Figure 14:**
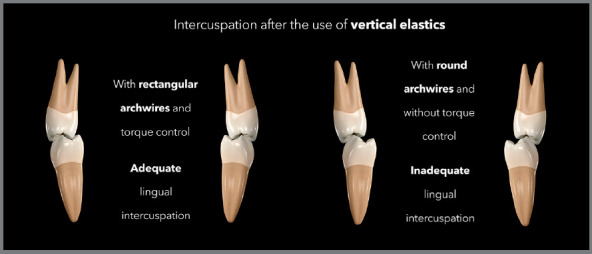
Illustration demonstrating the intercuspation performed with vertical elastic and rectangular wires, with torque control, promoting correct buccal and lingual contacts at the end of the treatment; and the intercuspation performed with vertical elastic and round wires, promoting torque change and lack of contact in the lingual region after treatment.

The most common side effect that occurs as a result using vertical elastics is the change in torque, with movement of the crown to the lingual and roots to the buccal, due to the formation of a moment of force during the buccal force application in relation to the center of resistance of the teeth (Fig 13). As a result, there will be contact between the buccal cusps; however, intercuspation will be impaired by the lingual due to the distance of these cusps, as a result of an incorrect torque (Fig 14). Another side effect, when used unilaterally, may be the inclination of the occlusal plane (roll axis), which may be accompanied by lateral flattening of the arch and deviation of the midlines.

## CLASS I VERTICAL ELASTIC

**Figure 15:**
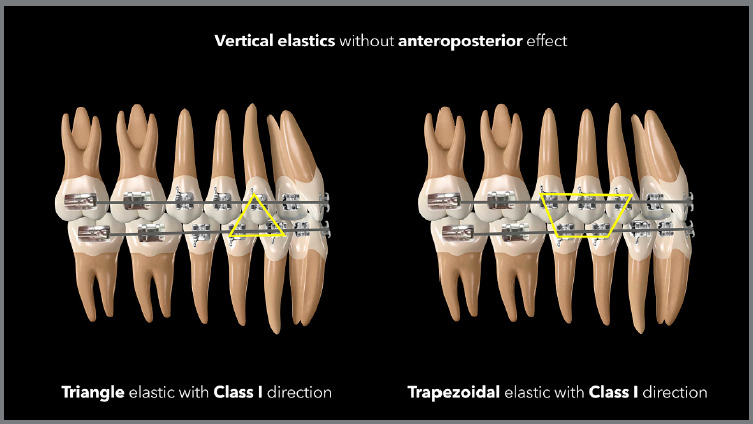
Illustration demonstrating two possibilities (triangular and trapezoidal) of vertical elastics with Class I orientation.

**Figure 16:**
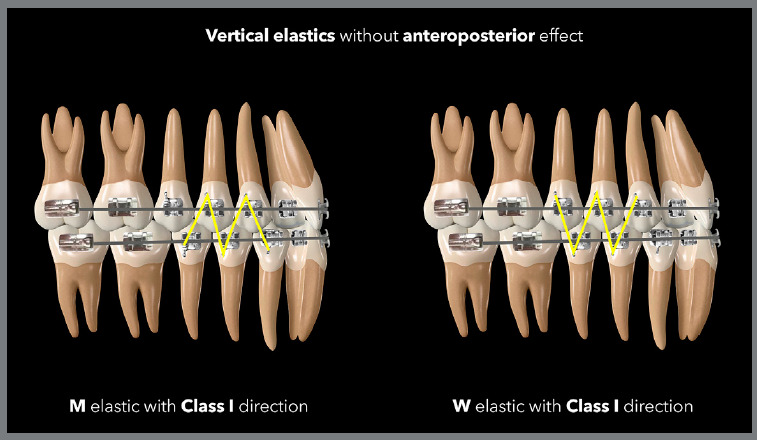
Illustration demonstrating two possibilities (M and W) of vertical elastics with Class I orientation.

**Figure 17:**
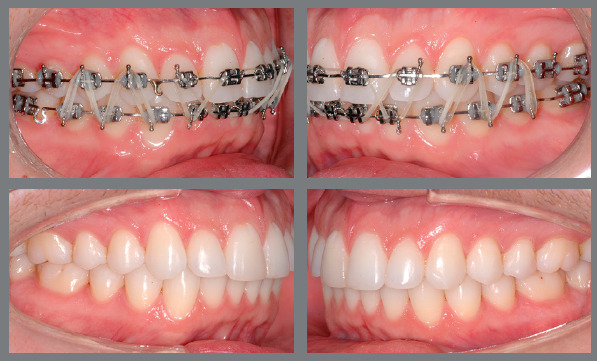
Patient treated with light force 3/8-in vertical elastics in an M-shape, in a case with a Class I anteroposterior relationship.

The Class I vertical elastic should only be used when extrusion effects are desired in the arches, without AP effects. The connection between the lower and upper arches should not form an AP resultant, and this normally occurs when the most mesial and distal connections of the elastic are in the same arch. Examples would be the cases of triangles formed by connecting the lower canines and first premolars with the upper canines, or M-shaped elastics, connecting three lower teeth with two upper teeth. In these cases, the distal and mesial connections are in the lower arch, without a sagittal component (Figs 15 and 16). Figure 17 shows a case in which M-shaped elastic was used in the final phase of the treatment.

## VERTICAL CLASS II ELASTICS

This elastic should be used when it is necessary to improve intercuspation in the vertical direction, associated with an improvement in the AP relationship between the arches, with a small posterior movement of the upper teeth, associated with a small anterior movement of the lower teeth. When the AP discrepancy is large, i.e., greater than 1 mm, it is recommended to first use a Class II AP elastic for sagittal correction, followed by a Class II vertical elastic, if necessary.

**Figure 18:**
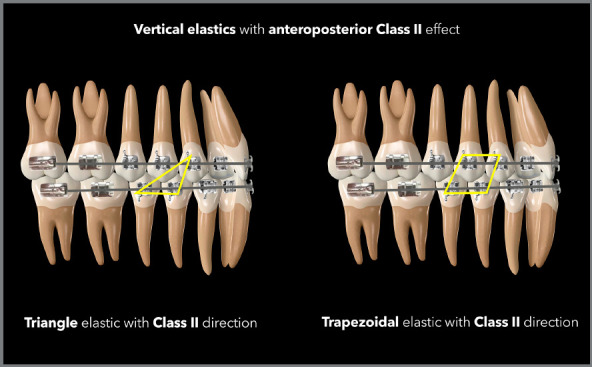
Illustration demonstrating two possibilities (triangular and trapezoidal) of vertical elastics with Class II orientation.

**Figure 19:**
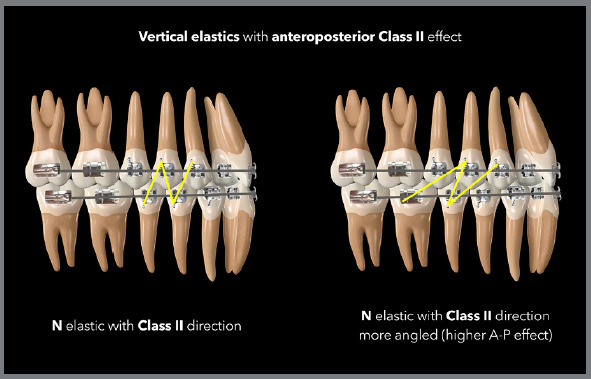
Illustration demonstrating two possibilities (N and N more angled) of vertical elastics with Class II orientation.

**Figure 20:**
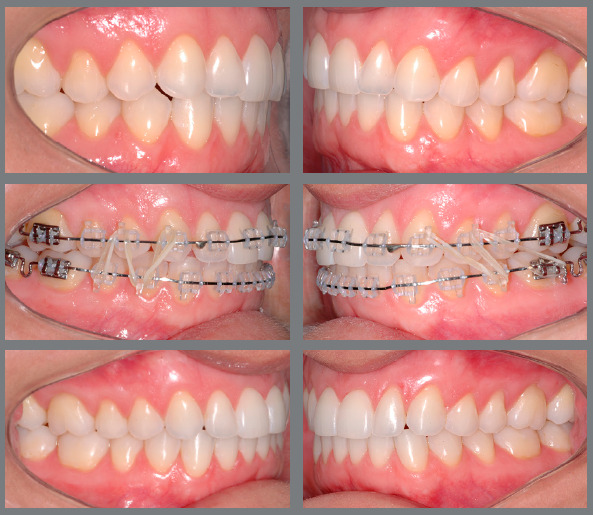
Patient treated with 5/16-in vertical elastics of light force in the shape of an N, for intercuspation and with orientation for Class II correction, being more angled on the left side, where there was a greater need for anteroposterior correction.

The most used shapes in these situations are triangles or trapezoids of 3/16-in or 1/4-in elastic bands of medium force, with a more distal connection in the lower arch and a more mesial connection in the upper arch, or light or medium force 5/16-in elastics in N shape, having inferior distal and superior mesial connections (Figs 18 and 19). The greater the need for AP correction associated with vertical correction, the greater the angulation of the elastic band, as shown in the case illustrated by Figure 20, in which there was a need for greater Class II correction on the left side - therefore, the elastic band on that side was used in a more angled manner. 

## VERTICAL CLASS III ELASTICS

The indication for using the vertical elastic with Class III direction is similar to those of Class II, with connection inversion, when we need the connection more distal in the upper arch and more mesial in the lower arch, distributing vertical force associated with anterior force in the upper arch superior and posterior force in the lower arch. 

**Figure 21:**
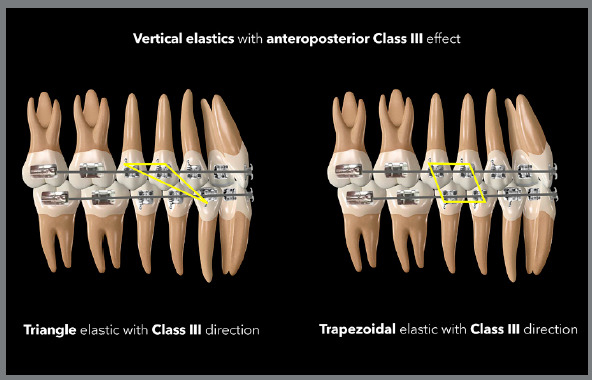
Illustration demonstrating two possibilities (triangular and trapezoidal) of vertical elastics with Class III orientation.

**Figure 22:**
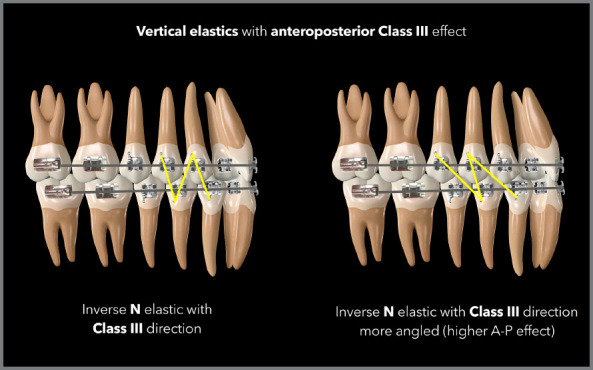
Illustration demonstrating two possibilities (inverse N and more angled inverse N) of vertical elastics with Class III orientation.

In this case, the same shapes and thicknesses of elastic used in Class II correction are normally used, as shown in the Figures 21 and 22. 

**Figure 23:**
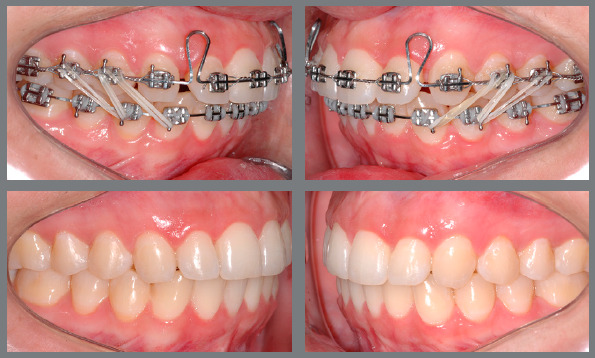
Patient treated with light force 5/16-in vertical elastics in a more angled inverse N shape, for intercuspation and with orientation for Class III correction, with improvement on the lower incisors position, obtaining overjet and allowing the upper spaces closure.

The case in Figure 23 demonstrates a situation of using 5/16-in elastics with light force with an N shape and angled to correct a small Class III relationship associated with an improvement in intercuspation.

## UNILATERAL VERTICAL ELASTICS

It is possible to use the vertical elastics unilaterally. However, elastics can only be used in this way when one really wants to correct inclination of the occlusal plane with extrusion of one or both arches on one side, as this will certainly be an expected effect in this configuration of use. Image 24 demonstrates a case in which the inclination of the upper occlusal plane was corrected using a miniscrew and, subsequently, the correction of the inferior arch on the same side was necessary. For this purpose, an M-shaped elastic was used unilaterally after stabilization of the upper arch with the miniscrew.

## ANTERIOR VERTICAL ELASTICS

The vertical elastic can be used in the anterior region when it is desired to close an open bite by means of extrusion of the upper and lower teeth. Due to the direct overload on the anterior teeth, there is an increased risk of root resorption, and its use is recommended only in situations of finishing and closing small anterior openings of a maximum of 2 mm.^5^ When square or rectangular-shaped elastics are used in the anterior region, there is a tendency for greater extrusion in the region of the lateral incisors and less in the region of the central incisors, forming a curvature in the arch. Therefore, it is suggested that it should be used in a format that applies similar force in all the regions of the anterior teeth, favoring the uniform closure of the bite, as shown in the case of Figure 25.

[Fig f24]

**Figure 24: f24:**
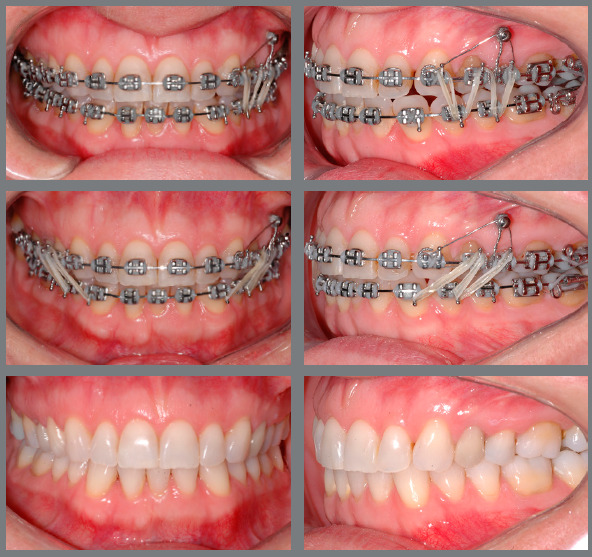
Patient treated with 3/8-in vertical elastics of light force in a W-shape, for unilateral extrusion, after correction of the cant of the upper occlusal plane with intrusion supported by a miniscrew. After leveling the lower arch, the patient started to use bilateral 5/16-in vertical elastics of light force with Class III direction (inverse angled N), for finishing procedures.

**Figure 25:**
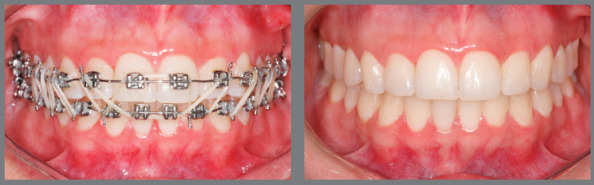
Patient treated with 3/8-in vertical elastics of light force in the anterior region, to improve the overbite.

## ELASTICS WITH TRANSVERSE DIRECTION

The intermaxillary elastics can also be used with transverse direction in situations in which there is a need to correct crossbite, buccal crossbite (Brodie bite), or even small midline deviations.

## ANCHORAGE PREPARATION

In situations of conventional crossbite or buccal crossbite correction, anchorage preparation will depend on the need for control or acceptance of side effects. In the arch in which greater control is desired, the use of a rectangular stainless steel archwire is recommended, whereas in the arch in which greater effect is desired, a thinner archwire can be used, allowing greater movement. In situations of midline correction, the use of rectangular arches is also recommended, with the option of reducing the caliber for greater effect on one of the arches, but with a great risk of side effects such as incorrect angulation of the teeth at the end of the mechanics with the elastics.

## CONNECTION

Regarding the use of elastics for correction of crossbites or buccal crossbites, it is possible to use buttons for lingual bonding, whereas the buccal connection can be made directly to the brackets, in clipped or welded hooks, or in bends in the wires such as omegas, deltas, or loops.

The connection for midline correction elastics must be through welded or clipped hooks. In these situations, the resultant of forces in the horizontal direction should be prioritized; therefore, make the upper hook downwards and the lower hook upwards, if the overbite allows.

## SIZE, FORCE, AND WEAR TIME

In cases of crossbite correction, the choice is usually the 1/8-in medium force elastic, and the heavy force elastic can be used if the former is not successful. As with other intermaxillary elastics, it is recommended to be used throughout the day, being removed only for feeding and hygiene. In cases of crossbite, it is common to need to use a bite plane to allow uncrossing and to prevent the elastic from being cut by the occlusal contact between the upper and lower teeth.

In cases of midline correction, the elastic of choice is usually the 3/16-in medium force, distributing around 100gf. It is important to note that this elastic band should not be used for a long period of time during treatment, as there is a risk of important side effects, as described below.

## SIDE EFFECTS

The most common side effects associated with crossbite elastics are extrusion and uneven tipping or torque of the supporting teeth. As a means of minimizing or eliminating these effects, a rectangular arch can be used, with or without control of second or third order bends or auxiliary devices such as a transpalatal bar or lingual arch.

**Figure 26:**
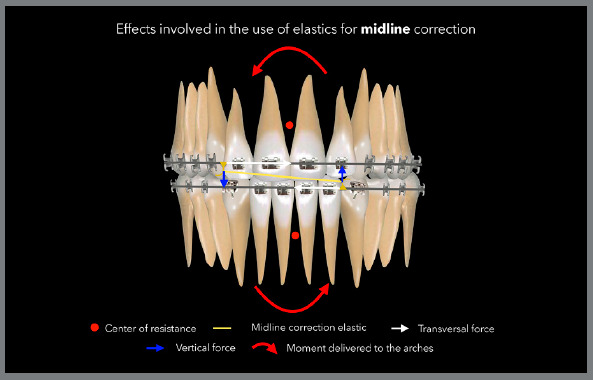
Illustration demonstrating the desired and undesired effects involved in biomechanics of midline correction with elastics.

**Figure 27:**
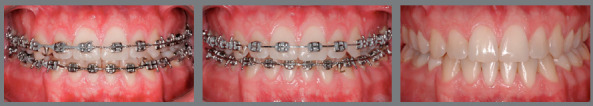
Patient treated with 3/16-in of medium force midline correction elastics connected to a hook initially fixed for occlusal direction in the lower arch and, later, being transferred to cervical, due to overbite gain.

With the use of midline correction elastics, the most common side effects are the cant of the occlusal plane (roll axis) due to the vertical component that forms during use, and incorrect angulation of the anterior teeth due to the application of a force that is more occlusal in relation to the center of resistance of anterior teeth.^28^ Again, rectangular archwires minimize these effects, allowing the midline to be corrected more efficiently by moving the body of the teeth, and not through angulation, which is normally very unstable after treatment, as the teeth tend to upright according to the apex, returning to the original position after the mechanics using elastics. The image in Figure 26 describes the side effects associated with the use of midline correction elastics, and in Figure 27, a case treated with midline correction elastics is presented.

## INTERMAXILLARY ELASTICS WITH CLEAR ALIGNERS

Intermaxillary elastics are also used in biomechanics with clear aligners, representing an important auxiliary resource in the correction of sagittal, vertical and transverse problems. Some studies have observed a limitation of aligners in large anteroposterior corrections, with movement of the whole arch. In these cases, it is therefore suggested to use higher force on the intermaxillary elastics or to use the elastics as an anchorage resource in sequential distalization biomechanics, with or without skeletal anchorage.^29^ ^-^ ^32^

## ANCHORAGE PREPARATION

Anchorage preparation for the use of intermaxillary elastics with aligners is different from that performed with a fixed appliance. As all teeth are involved from the beginning of the treatment by the aligner, the anchorage already exists from the beginning of the treatment and the elastics can be used from the first set of aligners.^31^

## CONNECTION

**Figure 28:**
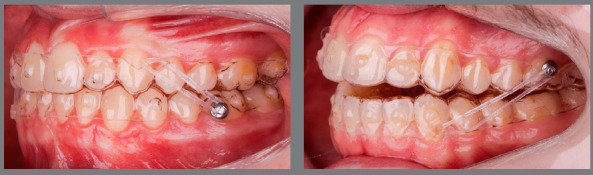
Intermaxillary elastics with anteroposterior direction with clear aligners, with posterior connection made with bonded buttons and anterior connection made direct to precision cuts in the aligners (Images provided by Dr. Guilherme Bernd).

The connection of intermaxillary elastics with the aligners can be performed in different ways, depending on the necessity in the biomechanics and preference of the professional. For elastics with Class II or Class III anteroposterior components, the connection can be made by means of precision cuts in the aligners, buttons bonded directly to the teeth or even in resin hooks, normally positioned in the regions of first molars and canines. In the posterior connection, the use of buttons bonded to first molars is recommended, as the aligners have little resistance at the ends, not being able to control the side effects of the elastics on the second molars.^32^ The connection in buttons, and not directly to the aligners, is also recommended in the posterior region, because the aligners show less retention in the posterior region and the elastic can promote the displacement of the aligners in the mandibular movements.^32^ In the anterior region, the aligners present greater stability to displacement, due to the use of anteroposterior elastics. Therefore, the connection in this region can be performed directly over the precision cuts in the aligners; however, the use of attachments positioned on the canines or over the adjacent teeth is suggested to increase retention and avoid any displacement of the aligners. Another option in the anterior region is also to use buttons bonded to the cervical region of the canines; however, in this situation there is a greater tendency to side effects on this tooth, such as rotation, inclination and extrusion. When these effects are desired to improve the position of canines, buttons can be used as an anterior connection (Fig 28).

## SIZE, FORCE, AND WEAR TIME

With the necessity for anteroposterior effect (Class II and Class III), a smaller elastic band than the one used with the fixed appliance must be used, because in the case of aligners the connection is in the region of the first molar and canine; therefore, with a smaller distance between the connection points. In these cases, a 3/16-in medium-strength or heavy-strength elastic is usually used.

For crossbite, the 1/8-in elastic band of medium strength is recommended; and for vertical improvements, the same elastics used with the fixed appliance are recommended, with the size, direction and force depending on each occlusal situation, as in fixed mechanics.

## SIDE EFFECTS

The side effects involved in biomechanics with aligners are similar to those seen with fixed appliances.

The point of posterior connection, in cases of Class II or Class III elastic use, can be on buttons bonded to first molars or second molars or even directly connected to precision cuts in the aligners. Posterior connections directly to the aligners are not recommended, as described above. In the traditional connection over the first molars, there is a tendency for these teeth to rotate, inclinate and extrude. The extrusion is counteracted by the presence of the aligner, when well adapted. Rotation and inclination can be counteracted by the well-fitting aligner, a button placed in the mesial region of the buccal surface, associated with attachment in the distal region of the buccal surface.

In the anterior region, connections made directly to the aligners are less likely to have a side effect on the teeth and have a greater distribution of force over all teeth in the arch. Connections made directly on buttons bonded to the canines can promote rotation, inclination and extrusion of the teeth involved. These effects can also be reduced by incorporating attachments into the canines, when the buccal surface area allows the use of a cutout for bonding the button and attachment together.

When they are used for crossbite correction, there is a tendency for extrusion, which is normally counteracted by the well-adapted aligner. Another side effect is the change in the torque of the teeth involved, which again can be counteracted by attachments positioned in the buccal region of the upper tooth and buccal or lingual region of the lower tooth.

During the use of vertical elastics to close the bite or improve intercuspation, there is a tendency to incorporate buccal torque on these teeth, which can be controlled by planning lingual torque on the aligners associated or not with attachments on these teeth.

## OTHER SIDE EFFECTS

### ROOT RESORPTION

In addition to the side effects described above, within each conformation of intermaxillary elastics, we can expect other effects that should normally be controlled during treatment, such as root resorption - which normally occurs in maxillary anterior teeth associated with the use of Class II elastics, vertical elastics, or midline elastics. This is due to the nature of the constant force applied by the elastics on these teeth, associated with short periods of deactivation and activation during the day (back and forth movement), during the removal and replacement of the elastics.^7^ Therefore, the use of intermaxillary elastics should be avoided in patients with a history of root resorption or loss of bone support.

### ALLERGIC REACTIONS

Approximately 3% to 17% of the population have an allergic reaction to latex at some level, and may present with stomatitis, edema, erythematous oral lesions, and even respiratory reactions. In these cases, it is recommended to use elastics made with non-latex or silicone elastomers, which are marketed by some brands of orthodontic products.^10^ ^-^ ^13^ ^,^ ^33^

### TOOTH MOBILITY

The appearance of moderate mobility while using intermaxillary elastics can be considered normal, especially when vertical elastics are used. This is due to the thickening of the periodontal ligament and the extrusion movement of the tooth in relation to the alveolus. During the retention period, teeth normally regain stability in their final position. In situations of teeth with a history of mobility due to periodontal disease and loss of bone support, the use of elastics should be avoided, in order to prevent a greater commitment to these teeth.^24^

### JOINT OR MUSCLE PAIN

Some patients experience joint or muscle pain when using intermaxillary elastics, especially elastics that distribute asymmetric force to the arches, consequently generating non-equivalent forces on both sides of the musculature and temporomandibular joints.^7^ ^,^ ^14^ In these cases, if the pain is mild to moderate, it can usually be resolved by reducing the time of elastic use in the first few days, with a gradual increase until the ideal period of 24 h a day is obtained. In situations of severe and limiting pain, the use of elastic bands should be stopped and, if necessary, ice packs should be used twice a day in the affected muscle region, associated with muscle relaxants if the pain is a muscle pain, or anti-inflammatory drugs if there is a joint pain. In these patients, use can be resumed for verification and, if the pain persists, the use of elastics should be stopped, and alternative mechanics should be planned.

## FINAL CONSIDERATIONS

There is no doubt about the importance of intermaxillary elastics in orthodontic treatments, due to their wide versatility and possibility of biomechanical application. However, it is important to have a deep knowledge of the desired effects and of the possible side effects that are normally associated with mechanics using elastics, so that the results are obtained as desired and planned by the orthodontist.
